# Weighty data: importance information influences estimated weight of digital information storage devices

**DOI:** 10.3389/fpsyg.2014.01536

**Published:** 2015-01-08

**Authors:** Iris K. Schneider, Michal Parzuchowski, Bogdan Wojciszke, Norbert Schwarz, Sander L. Koole

**Affiliations:** ^1^Mind and Society Center, University of Southern CaliforniaLos Angeles, CA, USA; ^2^Department of Psychology, University of Southern CaliforniaLos Angeles, CA, USA; ^3^Department of Clinical Psychology, VU University AmsterdamAmsterdam, Netherlands; ^4^Psycholinguistique and Psychologie Sociale Appliquée, University of FribourgFribourg, Switzerland; ^5^Sopot Social Cognition Lab, University of Social Sciences and HumanitiesSopot, Poland

**Keywords:** weight, embodiment, importance, digital data, judgment, relevance, metaphor, social cognition

## Abstract

Previous work suggests that perceived importance of an object influences estimates of its weight. Specifically, important books were estimated to be heavier than non-important books. However, the experimental set-up of these studies may have suffered from a potential confound and findings may be confined to books only. Addressing this, we investigate the effect of importance on weight estimates by examining whether the importance of information stored on a data storage device (USB-stick or portable hard drive) can alter weight estimates. Results show that people thinking a USB-stick holds important tax information (vs. expired tax information vs. no information) estimate it to be heavier (Experiment 1) compared to people who do not. Similarly, people who are told a portable hard drive holds personally relevant information (vs. irrelevant), also estimate the drive to be heavier (Experiments 2A,B).

## INTRODUCTION

Imagine two identical USB-sticks. One holds the data files of four experiments with world-changing results, thus, very important data. The other holds a recipe for hard-boiled eggs, relatively unimportant data. Is there a difference in weight between the two USB-sticks? This question seems almost silly, and a conventional and straightforward answer to this question is ‘no’. Digital data storage depends on changes in magnetic polarities, with no influence on the perceptible weight of the data storage device itself. As such, the weight of a USB-stick does not change depending on the importance of information that is stored on it.

Nevertheless, when people talk about the information on the USB-stick, they may well use references to weight to indicate the difference in importance between the two. For instance, someone describing the relatively important content of the first USB-stick may refer to it as “weighty data” and emphasize that it is not to be “taken lightly,” whereas the recipe for hard-boiled eggs will not be considered a “weighty matter” by most people. Thus, people seem to use expressions relating to weight when they are trying to convey importance.

Importance and weight are closely associated in human experience, because a heavy weight signals that dealing with the object is more demanding (cf. Proffitt, 2008) and requires more planning as well as physical and cognitive effort (Jostmann et al., 2009). This pervasive human experience of a relationship between physical weight and psychological importance is reflected in language, where weight conveys importance even when actual weight is irrelevant (e.g., “Her opinion carries a lot of weight”). The relationship between weight and importance in metaphors may reflect the way people conceptualize the concept of importance (cf. Lakoff and Johnson, 1999; Landau et al., 2010; IJzerman and Koole, 2011; Lee and Schwarz, 2012, 2014; Slepian and Ambady, 2014).

According to theories of grounded cognition, physical sensory experiences are part of how people represent and understand abstract concepts. As a result, induction of physical experiences associated with a concept can activate the concept as a whole (Barsalou, 1999, 2008; Lakoff and Johnson, 1999). For instance, when people are holding a heavy clipboard compared to a light clipboard, they find societal issues more important (Jostmann et al., 2009; Maglio and Trope, 2012), heavier books are considered more influential than lighter books (Chandler et al., 2012; IJzerman et al., 2013), and carrying a heavy shopping bag causes people to judge nutritional information on packaged food as more important (Zhang and Li, 2012).

Because physical experiences are part of people’s abstract representations of concepts, the activation of an abstract concept can also activate the physical experience tied to this concept through simulation (Barsalou, 1999, 2008). Indeed, this influence of activation of abstract concepts on physical experiences has been found for a range of domains. For instance, not only does warmth induce feelings of interpersonal closeness (IJzerman and Semin, 2010), but feelings of loneliness can make people feel colder (Zhong and Leonardelli, 2008; IJzerman et al., 2012; Szymkow et al., 2013). Similarly, social suspicion can influence odor perception (Lee and Schwarz, 2012) and attitudinal ambivalence can lead people to make wavering movements (Schneider et al., 2013). Given that weight and importance are closely connected in people’s representation, and weight can activate importance, the question becomes whether importance can also influence people’s experience of weight. Because the feeling of weight is a ubiquitous experience in people’s lives, it is important to understand how abstract representations influence these seemingly objective perceptions.

Whereas the influence of weight on judgments of importance has received considerable attention, the reverse influence of importance on judgments of physical weight has not. Moreover, the scarce findings are not conclusive. For instance, in one experiment, people were asked to think about past important decisions (i.e., activation of importance) after which they judged the weight of a product package, both on a subjective (perceived heaviness) as well as an objective (grams) measure of weight. Here, importance activation increased both judgments, but the increase did not significantly differ from baseline (Zhang and Li, 2012; Experiment 5). Conceivably, importance did not influence weight estimates strongly because it was not related to the object being judged.

Other work, where importance information was directly related to the object, did find statistically significant differences in weight judgments as a result of importance information. When participants were asked to estimate the weight of a book, those who thought the book was important estimated the book to be heavier than those who did not think it was important (Schneider et al., 2011). However, one could argue that for books, the degree of importance conveys at least some anecdotal information about weight. Important books (e.g., textbooks) are more often printed on heavier paper of better quality, and have hardcovers, rendering them heavier. Vice versa, books made of low quality paper are considered so inconsequential that “pulp fiction” is synonymous with low quality writing (Merriam-Webster online, nd). Thus, one could argue that participants’ perceptions of the weight of books are not based on importance information *per se*, but on latent information about the materials typically associated with important books.

The aim of the current work was twofold. First we wanted to investigate whether importance information influences weight judgments, beyond specific objects (i.e., books), to test the effects’ generalizability across targets of judgment. Ideally, there should be no relationship between the weight of the physical object and the importance of its content. One class of objects that meets this criterion is data storage devices. In data storage devices, information is stored in a digital format that bears no direct relation to its physical dimensions (i.e., zeros and ones reflected in magnetic states). A USB-stick that contains the data of experiments with world changing results does not differ in its perceptible physical properties from a USB-stick that contains a text file with a recipe for hard-boiled eggs. Furthermore, the importance of the stored information is not represented in the physical properties of the storage device, in contrast to books, which may come with or without hard covers, thick paper, and other properties that people associate with important books. Hence, data storage devices provide a particularly compelling object category for testing the idea that subjective importance may influence estimates of physical weight. Second, we wanted to conceptually replicate previous findings (Schneider et al., 2011) in order to better understand the robustness of the relationship between importance and weight estimates.

We conducted three experiments to investigate the influence of importance information on weight estimates of data storage devices. In all studies, we report all manipulations, all participants and exclusions, and all dependent measures. Sample sizes are the result of collecting as many participants as possible within the data collection period. We analyzed the results using IBM SPSS Statistical software package, and calculated *Cohen’s* effect sizes *d*_s_ (standardized mean difference between two groups of independent observations, Cohen, 1988) using a calculation sheet developed by Lakens (2013).

## EXPERIMENT 1

In Experiment 1 we used a generic USB-stick (Kingston 4GB USB-stick, 12 g), and manipulated the alleged importance of the information stored on the drive. We added a control condition in which the USB-stick purportedly contained no information. We hypothesized that people who thought the information on the USB-stick was important would give higher weight estimates compared to people who thought the information on the USB-stick was unimportant, or who thought there was no information on the USB-stick. The same formatted empty USB-stick was used in all conditions, thus only the information about the USB-stick differed.

### METHOD

#### Participants and design

Seventy-five students from the Gdansk’s Sport Academy (38 females, *M*_age_ = 25.82, SD_age_ = 3.19) voluntarily participated and were randomly assigned to one of three conditions (important information, expired information, no information). Estimated weight of a USB-stick served as main dependent variable.

#### Procedure and materials

A female experimenter approached participants on campus. After introducing herself, the experimenter explained to the participants that she was conducting an experiment about judgments of everyday objects and invited them to participate. It was made clear that the participant could quit the experiment at any time. Questions were administered orally and responses were recorded by the experimenter.

After participants agreed to participate, the experimenter registered participant’s sex, age, and study major. Participants were then given a USB-stick to hold in their hand and asked to indicate whether they had recognized the object they were holding. The response scale ranged from 1 (not at all) to 4 (definitely). Next, the experimenter said, “*Now I will tell you a bit more about the object you are holding. This is a flash drive, also called a USB-stick – it is a data storage device. This one was made in Japan and was manufactured in 2011. It has 4 GB of storage space.”*

After this general introduction, we manipulated importance information. In the important condition participants were told, *“This USB-stick is full of fiscal data. It stores the archive of all tax history from the last 2 years of a major company from this region. These documents are used for the daily functioning of this company.”* In the expired condition participants were told, *“This USB-stick is full of fiscal data. It stores the archive of all tax history from the year 1999–2000 of a major company from this region. These documents are now expired.”* In the no information condition participants were told, *“This USB-stick has been recently formatted, which means that it is an empty storage – it does not hold any data.”*

After this short description, participants were asked to indicate how important they thought the data stored on the USB-stick was on a scale from 1 (definitely not important) to 4 (definitely important) and what the weight of the USB-stick was in grams^.^ As an exploratory measure we also included a measure of size of the USB-stick and asked participants to sketch the size and shape of the USB-stick in a designated area on the experimenter sheet. We calculated the area of the sketches of the USB-stick by multiplying width and height of the drawing in millimeters. Finally, participants were thanked and debriefed.

### RESULTS AND DISCUSSION

Three participants were removed from the dataset because their scores on the weight judgment were more than 3 SD removed from the mean. Weight estimates of these participants were 80, 100, and 100 g, respectively, while the mean was 10.60 g and both the mode and median were 5 g. This left 72 participants in the dataset (27 in the important information, 22 in the expired information condition, and 23 in the empty condition), of which 36 were female.

#### Manipulation check

We first checked whether participants were familiar with USB-sticks. In general, participants indicated that they had recognized the object (*M*_grand_ = 3.80, SD_grand_ = 0.40) and there were no pre-manipulation differences between the experimental groups, *F* < 1. A one-way ANOVA on importance ratings revealed that judgments of importance differed between conditions, *F*(2,69) = 98.48, *p* < 0.001, $\eta_{p}^{2}$ = 0.74. More specifically, *post hoc* tests showed that the important information was judged more important (*M* = 3.85, SD = 0.60) than the expired information (*M* = 2.09, SD = 0.68), *p* < 0.001, *Cohen’s d*_s_ = 2.76, 95% CI_difference_ [1.31,2.22], which in turn was judged to be more important than no information (empty USB-stick, *M* = 1.30, SD = 0.70), *p* = 0.001, *Cohen’s d*_s_ = 1.14, 95% CI_difference_ [0.31,1.26]. Thus, our manipulation of importance was successful.

#### Weight

A one-way ANOVA revealed that, as expected, there was a statistically significant difference between the estimated weight between conditions, *F*(2,69) = 33.41, *p* < 0.001, $\eta_{p}^{2}$ = 0.49. More specifically, *post hoc* tests showed that the USB-stick containing important information was judged heavier (*M* = 11.19, SD = 3.33 g) than the one containing expired information (*M* = 4.86, SD = 3.56 g), *p* < 0.001, *Cohen’s d*_s_ = 1.84, 95% CI_difference_ [4.09,8.55], as well as the USB-stick that held no information (*M* = 4.61, SD = 2.79 g), *p* < 0.001, *Cohen’s d*_s_ = 2.12, 95% CI_difference_ [4.37,8.78]. The latter two did not differ from each other, *p* = 0.79, 95% CI_difference_ [-2.06, 2.57], suggesting that participants did not infer differences in weight from the presence of data *only*. Thus, participants perceived a USB-stick containing important information almost twice as heavy as a USB-stick containing unimportant information or no information (a relatively large effect, we return to this in Experiment 2A). Furthermore, the more important people thought the information on the USB-stick was, the heavier they thought the USB-stick was *r* = 0.61, *p* < 0.001 and the bigger they drew the area, *r* = 0.60, *p* < 0.001. The correlation between weight and area was also significant, *r* = 0.44, *p* < 0.001, showing that the heavier people thought the USB-stick was, the bigger they drew its area. These findings are in accordance with everyday experiences people have in the natural environment where heavier objects are usually larger (Ernst, 2009).

#### Size

A one-way ANOVA revealed that participants estimated the size of the USB-sticks differently, *F*(2,69) = 14.00, *p* < 0.001, $\eta_{p}^{2}$ = 0.29. Specifically, *post hoc* tests showed that the USB-stick containing important information was drawn as a bigger shape (*M* = 359.22, SD = 89.94 mm^2^) than the one containing expired information (*M* = 227.86, SD = 133.21 mm^2^), *p* < 0.001, *Cohen’s d*_s_ = 1.16, 95% CI_difference_ [65.18, 197.54] as well as the empty USB-stick (*M* = 197.74, SD = 123.86 mm^2^), *p* < 0.001, *Cohen’s d*_s_ = 1.49, 95% CI_difference_ [ 96.10, 226.86]. The latter two did not differ from each other, *p* = 0.39, CI_difference_ [-38.59, 98.84].

In sum, Experiment 1 shows that people who think there is important information on a USB-stick, also think it is heavier compared to people who think there is expired information or no information on the USB-stick, providing a conceptual replication of previous findings (Schneider et al., 2011).

## EXPERIMENT 2A

The findings in Experiment 1 show that importance information can influence judgments of weight when people think the information is important to a large company or not. However, in everyday life, people are more likely to carry around their own information on their data storage device. Thus, in Experiment 2A, we set out to replicate the findings in Experiment 1 using a more ecologically valid manipulation of importance. To do so we made information stored on a portable hard drive (TOSHIBA Store Alu 2S 500 GB, 181 g) personally relevant or irrelevant to participants, by either telling them it was a detailed compendium of courses in their own university and subject (Psychology at the University of Social Sciences and Humanities, Campus in Sopot) or for courses in another university and other subject (Economy at the Gdansk School of Banking). The same formatted, empty portable hard drive was used in both conditions, thus only the information about the hard drive differed.

Additionally, in Experiment 1, the effect size for the weight estimates was particularly large. Possibly this was due to the fact that participants *first* indicated how important they thought the information was and *then* estimated the USB-stick’s weight. This order might have made importance more salient, which could have enhanced our manipulation. Thus, in Experiment 2A we asked participants to *first* estimate the weight of the portable hard drive and *then* asked about the perceived importance of the data that was on it. Finally, because in Experiment 1 some participants had made extreme weight judgments (and were removed from the data set) we now also included an anchored measure of weight using a line scale to limit the range within which weight could be estimated, in hopes of preventing outliers.

### METHOD

#### Participants and design

Fifty students (30 females, *M*_age_ = 22.64, SD_age_ = 1.17) voluntarily participated. The experiment followed a one-factor design with two between-subjects conditions (importance: personal relevance vs. personal irrelevance) and estimated weight of a portable hard drive served as the main dependent variable.

#### Procedure and materials

Participants were approached on campus. The experimenter introduced the experiment and recorded age, sex, and study major and whether participants recognized the object they were holding, in the same manner as in Experiment 1. After participants agreed to participate, the experimenter introduced the object by saying: “*It’s a 20 GB portable hard drive. Data stored on this drive includes Word and PowerPoint files and photographs of materials and notes from almost all courses gathered by students from University of Social Sciences and Humanities, Campus in Sopot (personally relevant condition)/Gdansk School of Banking (personally irrelevant condition).*”

After this short description, participants were asked to indicate how much they thought the portable hard drive weighed on two measures. First, they indicated weight by indicating a spot on a 100 mm line anchored on the left side by *“10 g”* and on the right side by *“1 kg”* Second, participants provided a numeric estimate of the weight in grams. Third, participants were asked to indicate how important they thought the data stored on the portable hard drive was on a scale from 1 (definitely not important) to 4 (definitely important). Finally, participants indicated how much they thought the portable hard drive was worth in Polish Zloty (PLN). To assess common user knowledge about data storage we also asked participants whether they had recognized the object, how much space was required for an average quality mp3 of 3 min on a 5-point scale ranging from 0.5 to 150 MB and for an average quality movie in.avi format ranging from 7 to 700 MB. Participants then indicated how certain they were about their answers on a four point scale ranging from 1 (not at all) to 4 (very confident). In general, participants indicated that they had recognized the object (*M*_grand_ = 4.86, SD_grand_ = 0.40) and there were no statistically significant differences on between our manipulation groups, *p* = 0.23. Most participants (65.3%) thought an average quality mp3 of 3 min would take up 5MB space and an average quality movie in avi format would take up 700 MB, indicating common user knowledge of data storage. Participants were rather confident about their answers (*M*_grand_ = 2.98, SD_grand_ = 0.82) and there were no statistically significant differences between the experimental groups, *p* = 0.22. After this, participants were thanked and debriefed.

### RESULTS AND DISCUSSION

One participant was removed from the dataset because s/he provided a weight judgment (line scale) more than 3 SD above the mean. The estimate of this participant was 870 g on the line scale, while the mean was 369.40 g, the median was 385 g, and the modal estimate was 390 g. This left 49 participants in the dataset (22 in the control condition), of which 30 were female.

#### Manipulation check

In general, participants indicated that they had recognized the object (*M*_grand_ = 4.86, SD_grand_ = 0.40) and there were no pre-manipulation differences between experimental groups, *p* = 0.23. An independent *t*-test on importance ratings revealed that participants in the importance condition thought the information on the portable hard drive was more important (*M* = 3.37, SD = 0.63) than participants in the control condition (*M* = 2.55, SD = 0.80), *t*(47) = 4.04, *p* < 0.001, *Cohen’s d*_s_ = 1.16, 95% CI_difference_ [0.41,1.24]. As such, our manipulation of importance was successful.

#### Weight

Because the correlation between the line-scale and the gram estimates was moderate (*r* = 0.51), we report findings for both measures separately. An independent *t*-test on the line scale of weight (see **Table 1** for means) indicated that participants in the personal relevance condition thought the portable hard drive was heavier than participants in irrelevance condition, *t*(36.45) = 2.39, *p* = 0.022, *Cohen’s d*_s_ = 0.69, 95% CI_difference_ [10.93,134.59]. The same was true for weight judgments in grams, *t*(47) = 1.75, *p* = 0.088, *Cohen’s d*_s_ = 0.50, 95% CI_difference_ [-11.73,164.96], although this test failed to reach statistical significance by conventional standards. When we converted scores on both measures to z-scores and ran them as a within-factors subject factor, we found only a main effect of importance, *F*(1,47) = 5.91, *p* = 0.019, but no interaction between condition and measure, indicating there was no systematic difference in pattern between the two measures.

**Table 1:** Experiment 2A, Mean Weight Estimates (SD) in grams for personally relevant vs. personally irrelevant information for line scale and numeric estimate.

	Relevant	Irrelevant
Line scale	401.85 (84.72)	329.10 (120.91)
Numeric estimate	298.89 (151.11)	222.27 (155.10)

#### Value

An independent *t*-test on value of the portable hard drive indicated that participants in the importance condition thought the portable hard drive was more valuable (*M* = 173.63, SD = 93.41) than participants in the control condition (*M* = 103.18, SD = 45.68), *t*(39.31) = 3.23, *p* = 0.001, *Cohen’s d* = 0.93. However, there was no correlation between value and weight, *p* = 0.22 and *p* = 0.18 for the line scale and gram estimate, respectively, nor between importance and value, *p* = 0.72.

In sum, Experiment 2A shows that participants perceived the same hard drive as heavier when they thought that it contained self-relevant information (important) than when they thought it contained information that was not self-relevant information (unimportant). In accordance with our intuition that the question order may have boosted the effect size in Experiment 1, the effect size in Experiment 2A was considerably smaller. Last, important objects were also deemed more valuable, but value judgments were not correlated with estimates of weight, mimicking previous findings that did not find a relationship between the importance and value of books (Schneider et al., 2011).

## EXPERIMENT 2B

### METHOD

To test the robustness of our effect, we repeated Experiment 2A using the same materials, procedure, and design, with exception of the value estimates, which were not included. Eighty psychology students (53 females, *M*_age_ = 23.04, SD_age_ = 4.38) voluntarily participated.

### RESULTS AND DISCUSSION

One participant was removed from the dataset because s/he provided a weight judgment (line scale) more than 3 SD above the mean. The weight estimate of this participant was 850 g while the mean was 331.19 g, the median was 330 g, and the mode was 210 g. This left 79 participants in the dataset (37 in the personal irrelevance condition), of which 52 were female.

#### Manipulation check

Again, participants had recognized the object (*M_grand_* = 4.19, SD*_grand_* = 1.34) and there were no pre-manipulation differences between experimental groups, *p* = 0.50. The manipulation was again successful, participants in the personal relevance condition thought the information on the portable hard drive was more important (*M* = 2.95, SD = 0.80) than participants in the control condition (*M* = 2.53, SD = 0.81), *t*(76) = 2.33, *p* = 0.022, *Cohen’s d*_s_ = 0.53, CI_difference_ [0.06, 0.79].

#### Weight

Because the correlation between the line-scale and the gram estimates was moderate (*r* = 0.56), we report findings for both measures separately. An independent *t*-test on the line scale of weight indicated that participants in the personal relevance condition estimated the portable hard drive to be heavier than participants in the personal irrelevance condition (see **Table 2** for relevant means) on both the line scale, *t*(77) = 1.75, *p* = 0.084, *Cohen’s d*_s_ = 0.34, CI_difference_ [-0.07, 1.01], as the gram estimates, *t*(70.12) = 1.92, *p* = 0.059, *Cohen’s d*_s_ = 0.42, CI_difference_ [-2.21, 116.78], although these tests failed to reach significance by conventional standards. In conclusion, the results of Experiment 2B, a close replication of Experiment 2, further support the idea that importance information influences weight estimates. When we converted scores on both measures to z-scores and ran them as a within-factors subject factor, we found only a main effect of importance, *F*(1,77) = 4.25, *p* = 0.043, but no interaction between condition and measures, indicating there is no systematic difference in pattern between the two measures.

**Table 2:** Experiment 2B, Mean Weight Estimates (SD) in grams for personally relevant vs. personally irrelevant information for line scale and numeric estimate.

	Relevant	Irrelevant
Line scale	356.79 (134.48)	309.46 (101.07)
Numeric estimate	177.02 (160.44)	119.74 (101.24)

#### Joint analysis of Experiments 2A,B

Although Experiment 2B showed similar results as Experiment 2A, the patterns failed to reach conventional levels of significance in both studies. To address this concern, and because the two studies were nearly similar, we followed Rosenthal’s (1978) suggestion for the combined analysis of independent studies, using the method of adding *z*’s. Specifically, we converted the one-sided *p*-levels of each experiment to a *z* score while retaining the direction of each experiments outcome. We then summed the *z*-scores and decided them by the square root of the number of studies and using this number obtained a combined significance level. These analyzes revealed a statistically significant effect of importance on the line scale, *combined z* (2.84), *p* = 0.005, as well as in grams, *combined z* (2.54), *p* = 0.011, across experiments.

## GENERAL DISCUSSION

The current work shows people’s estimate of the weight of a data storage device is influenced by the degree to which they think the object is important, even though the importance of an object is not objectively associated with its weight. In three studies, importance information systematically influenced the weight estimates of data storage devices. Specifically, people who thought the information on a data storage device was important for the tax administration of a major company (Experiment 1) or was self-relevant by means of pertaining to their own course work (Experiments 2A,B) estimated the devices to be heavier than people who thought that the information on the device was expired (Experiment 1), not self-relevant (Experiments 2A,B), or completely absent (Experiment 1). Combined with other work (Schneider et al., 2011) the present work adds to previous findings that importance information systematically influences estimates of an object’s weight, proving more insight in the generalizability of the types of objects affected and the robustness of the relationship, providing a base from which possible mechanisms can be further explored.

This work constitutes a notable extension of the literature on weight perception. Previous work has shown that weight perceptions are influenced by the physical properties of an object. Lighter colored objects are judged to be heavier when picked up than darker colored objects (i.e., Walker et al., 2010), smaller objects seem heavier than larger objects (size–weight illusion, first found by Charpentier, 1891, but for a translation see Murray et al., 1999), and a tetrahedron (pyramid shape) seems heavier than a cube (Kahrimanovic et al., 2010). Even psychological states, such as the feeling of being in power can influence perceptions of weight (Lee and Schnall, 2014). However, this work shows that psychological variables *attached to an object*, such as importance, reliably influence how people perceive its weight (also see Schneider et al., 2011).

It seems unlikely that the observed effects of importance on weight estimates were merely due to demand characteristics. First, we took care to never mention “importance” or related words in our manipulations. However, the effect size in Experiment 1, where importance was relatively salient, seemed larger than the effects in Experiments 2A and 2B. Conceivably this is related to the salience of importance as a result of an explicit item asking about importance preceding the weight estimates, suggesting that explicitly mentioning importance may influence weight perceptions. Nevertheless, even with a more subtle approach, we observed an influence of importance information on weight estimates.

In our research, we asked people to orally report their estimates of weight. We assume that they report their physical experience of weight. Nevertheless, one may argue that people do not *really* experience a sense of weight, but instead, report numbers that are higher based on a semantic association. Previous work showed that importance influenced estimates of weight, but not of retail prize (Schneider et al., 2011). In our findings here, we found that value was estimated higher in the important conditions, but does not seem related to importance ratings or weight estimates. As such, the relationship between importance and value, and even weight, remains elusive and warrants additionally investigations.

The current work provides more information about the breadth of the effect of importance on estimates of physical weight. An important next step is to further unpack possible underlying mechanisms that account for this relationship. One way could be to assess the muscular changes in people’s hands, in order to test whether there really is a “simulation” of weight, which might result in differences in grasp intentions for objects (cf. Liuzza et al., 2012) that hold content of differential importance. Indeed, it has been found that a mere label with the words “long” or “short” on an object can influence motor behaviors in relation to this object (Gentilucci and Gangitano, 1998). Possibly similar effects can be induced by abstract labels, such as “important” and “irrelevant” through their metaphorical association with weight (Zhang and Li, 2012). However, when the mechanism is not simulation, but a contextual effect of the weight-as-importance metaphor, then individual differences in metaphor use may be more informative (Fetterman and Robinson, unpublished manuscript). Arguably, these possibilities are not mutually exclusive (Slepian and Ambady, 2014), and exploring the relative conditions of their operation may be more fruitful.

Apart from importance, other types of information may also influence the perceived weight of units that lack perceptible weight. For instance, few people have ever touched a calorie but anecdotal evidence from the food and beverage industry suggests that an absence of, or smaller amounts of certain units (e.g., calories, alcohol, nicotine), is sometimes referred to as light. Future research might investigate whether people experience “light” products as weighing less and appearing smaller than high-caloric foods. To get around self-reported values, these research questions could be investigated by using measures that go beyond self-report, such as grasping behaviors (Liuzza et al., 2012) or picking a box that would fit the object. Investigating whether perceptually heavy versus light foods are perceived to have more or less calories may have far-ranging implications in the field of marketing and consumer research.

The finding that important objects were also perceived to be bigger raises the question whether the opposite also occurs: do larger objects seem more important? If so, this may have interesting applied implications. For instance, producers of digital storage devices, looking to convey that their devices are suitable for storing important information, may not benefit from decreasing the device’s size and weight. If people feel that important information has more weight, they may prefer to store it on more substantial devices. We may therefore prefer smaller and less weighty devices to store lighthearted entertainment, but may want to keep the videos of our parents living overseas and the data files of world changing experiments on something big and heavy.

## AUTHOR CONTRIBUTIONS

Michal Parzuchowski and Iris K. Schneider developed the experimental operationalization and design, analyzed and interpreted the data. Michal Parzuchowski performed data collection. Iris K. Schneider wrote the first draft of the manuscript. Sander L. Koole, Bogdan Wojciszke, Norbert Schwarz, and Michal Parzuchowski participated in drafting the article and made critical revisions. All authors approved the final version of the paper.

## Conflict of Interest Statement

The authors declare that the research was conducted in the absence of any commercial or financial relationships that could be construed as a potential conflict of interest.
